# Effects of environmental factors on dengue incidence in the Central Region, Burkina Faso: A time series analyses

**DOI:** 10.1371/journal.pntd.0013356

**Published:** 2025-07-28

**Authors:** Jean Claude Romaric Pingdwindé Ouédraogo, Sylvain Ilboudo, Richard Joshua Tetteh, Charles Kyei, Siaka Lougué, Wendlasida Thomas Ouédraogo, Salfo Ouédraogo, David Dosoo, Kwaku Poku Asante, Léon Gueswendé Blaise Savadogo

**Affiliations:** 1 Laboratoire de Développement de Médicament (LADME), Université Joseph Ki-Zerbo, Ouagadougou, Burkina Faso; 2 Département de Médecine Traditionnelle, Pharmacopée et Pharmacie (MEPHATRA-Ph), Institut de Recherche en Sciences de la Santé (IRSS)/CNRST, Ouagadougou, Burkina Faso; 3 Kintampo Health Research Centre (KHRC), Kintampo, Ghana; 4 Département de Biologie Médicale, Santé Publique (BioMed/SP), Institut de Recherche en Sciences de la Santé (IRSS)/CNRST, Ouagadougou, Burkina Faso; 5 Direction Régionale de la Santé (DRS) du Centre, Ouagadougou, Burkina Faso; 6 Institut Supérieur des Sciences de la Santé (INSSA), Université Nazi Boni, Bobo-Dioulasso, Burkina Faso; University of Virginia School of Medicine, UNITED STATES OF AMERICA

## Abstract

**Background:**

Dengue is endemic in Burkina Faso with sporadic outbreaks during the decade 2011–2021. Dengue control depends on the ability to predict future outbreaks. This study aimed to forecast dengue cases using historical data between 2016 and 2021.

**Methods:**

The study covered the Central Region, Burkina Faso, with dengue monthly data from the National System of Health Information (SNIS) and environmental data from the National Agency of Meteorology (ANAM). The Autoregressive Distributed Lag (ARDL) model was performed to forecast dengue cases between 2022 and 2025.

**Results:**

Dengue cases increased gradually between 2016 and 2021, with seasonal spikes during the year. The 95 per cent confidence interval exceeds 5000 cases by 2023 and reaches about 10,000 cases by 2025. From the ARDL results, the lagged variable Dengue cases (-1) showed a strong positive association (coefficient = 0.76; p-value = 0.00) and the variable Dengue cases (-2) a negative association (coefficient = -0.47; p-value = 0.01). The Population statistically impacted dengue incidence (coefficient = 0.00; p-value of 0.01). Relative humidity (-1) and Relative humidity (-4) positively affected dengue cases (coefficient = 114.26; p-value = 0.00 and 90.84; p-value = 0.00 respectively). Furthermore, Rainfall (-4) had a negative influence on dengue incidence (Coefficient = -6.91; p-value = 0.00. D.Minimum temperature (-3) positively influenced dengue cases (Coefficient = 223.20; p-value = 0.01). D.Wind speed showed a negative relationship (Coefficient = -925.31; p-value = 0.02), while D. Wind speed (-3) had a positive relationship (Coefficient = 875.04; p-value = 0.02). In addition, the ARDL long-run results revealed a positive association between dengue cases and population size (p-value = 0.02), Relative humidity (p-value = 0.01), and D.Minimum temperature (p-value = 0.02), and a negative association with Rainfall (p-value = 0.04).

**Conclusion:**

Dengue cases are forecasted to increase in the Central Region between 2022 and 2025. It is then crucial to develop long-term interventions against dengue, integrated with interventions for other neglected tropical diseases.

## Author summary

Dengue fever is a growing global threat, partially due to unplanned urbanization and climatic change. Africa, which is increasingly paying the price, lacks comprehensive data on the disease. Dengue fever has increasingly threatening Burkina Faso in recent years with sporadic outbreaks. We therefore conducted this study in the Central Region of Burkina Faso to better understand its dynamics. According to previous data, dengue cases are expected to continue rising between 2022 and 2023. This was confirmed in 2023, as Burkina Faso declared the largest dengue outbreak ever recorded in Africa. Dengue fever transmission is modulated by climatic factors. Thus, recent weather conditions associated with rainfall, humidity, ambiant temperature and wind speed were found to influence the occurrence of new cases. The influence of climate on the emergence of dengue new cases is also remote. These results highlight the complex dynamics of dengue-climate interactions. In addition to the climate, the growing population of the Region will favour the occurrence of dengue new cases. These data have enabled us to gain a better understanding of the dengue situation in Burkina Faso.

## Background

Dengue is one of the most widespread vector-borne diseases, threatening about one-half of the world population [1]. The disease thrives in urban and periurban environments due to rapid urbanization and sanitation issues. Dengue is found in the tropical and sub-tropical zones, mostly in Asia, America and Africa. Data are available for Asia and America but lacking in Africa due to poor surveillance that underreports the cases [2–4]. However, the dengue burden in Africa is similar to that in America [4]. Moreover, 15 out of 47 countries of the WHO African Region reported dengue outbreaks in 2023, with 16,000 suspected cases and 8000 confirmed cases [5].

In Africa, East, Central and West Africa are the hotspots for dengue [6,7]. West Africa, including Burkina Faso, has been spotted as the new front for dengue [8]. Dengue was first reported in Burkina Faso in 1925 and 1982, and subsequent studies confirmed its endemicity and reported some epidemics [9–13]. As of March 3, 2024, the country reported a total of 164,848 suspected cases, including 73,497 probable and confirmed cases, with a case fatality rate of 0.45% (over suspected cases) [14].

Preventing and controlling dengue fever has, therefore, become a major challenge for the health systems as they can be rapidly overwhelmed by an unusual rise in cases. Passive surveillance data with environmental information can help in this respect through an early warning system. The early warning system for dengue aimed at rapid decision-making processes triggering intervention strategies in the event of a disease in order to minimize the impact on a specific population [15]. However, the early warning system for dengue fever is challenging due to the interactions between biological, environmental, vector and virus-related factors involved.

Dengue virus and *Aedes* vector lack thermostatic mechanisms, making them susceptible to environmental conditions [16–18]. Systematic reviews highlighted that temperature (95,2%), rainfall (81,0%) and relative humidity (77,4%) were the best predictors of dengue incidence, dengue epidemics and early warning systems of dengue epidemics, along with other factors such as wind direction and speed, and insolation [15,19,20]. However, data on dengue predicting factors are widely available for Asia and America and lack for Africa [19,20].

Since the 2013 outbreak, the passive surveillance of dengue has been improved in Burkina Faso, allowing to predict dengue incidence in the Central Region. Generalized Additive Models (GAM) established that minimum and maximum temperature, relative humidity, and wind speed have been associated with an increase in dengue cases in the same region between 2017 and 2019 [21]. If diverse models, such as machine learning or statistical models (linear regressions, Poisson or Generalized additive models) are used to predict dengue incidence, time series and autoregressive models are more commonly used [15,19,20,22].

This study aims to predict dengue incidence through time series analyses, using environmental data between 2016 and 2021.

## Methods

### Ethics statement

This research was approved by the national Ethics Committee for Health Research (CERS: “Comité d’Ethique pour la Recherche en Santé”) [N° 2022-12-257] and the Institutional Ethics Committee for Health Research (CEIRES: “Comité d’Ethique Institutionnel pour la Recherche en Santé”) of the Institut de Recherche en Sciences de la Santé (IRSS) [N° 032–2022/CEIRES].

### Study setting

Burkina Faso is organized into 13 regions, including the Central Region, which covers 2 826,28 square kilometres [23]. The Central Region comprises the capital city, Ouagadougou, and 6 surrounding rural municipalities: Koubri, Komsilga, Tanghin-Dassouri, Komki-Ipala, Pabré, and Saaba [24].

The region features a Sudano-Sahelian climate, characterized by alternating dry season lasting seven months (from October to May) and rainy season lasting five (05) months (June to September) [23,24]. Average annual temperatures vary between 17°C and 36°C. Rainfall reaches 750 mm/year, intermediate between the Sahelian climate zone in the north and the Sudano-Guinean climate zone in the south [23]. August tends to be the wettest month of the year. A total of 386.62 km from the Nakambé and Nazinon basins flow through the region [24].

In the Central Region, the health system consists of 5 health districts, which are Boulmiougou, Sig-Nonghin, Bogodogo, Baskuy, and Nongr-Massom districts. It includes several public and private health facilities and three university hospitals: University Hospital of Bogodogo (CHU-B), University Hospital of Tengandogo (CHU-T), and Yalgado Ouedraogo University Hospital (CHU-YO).

The Central region was the most populated of Burkina Faso in 2019, with 3,032,668 inhabitants [24]. The age distribution showed 62.35% adults (30.97% males and 31,38% females) and 31,38% children under 15 years.

### Data source

This study used the number of dengue cases and environmental data on a monthly basis for the Central Region. Demographic data were also used during the analyses.

Dengue monthly data aggregated through passive surveillance were retrieved from the online Health Data Depository of Burkina Faso (ENDOS-BF). Data included suspected, probable, and confirmed cases. Suspected cases were those diagnosed clinically by the presence of fever with headache, retro-orbital pain, myalgias, arthralgias, skin rash, bleeding manifestations, and/or shock syndrome. Cases with a positive rapid diagnostic test (Positive Antigen NS1 and/or Immunoglobulin M and/or Immunoglobulin G) were classified as probable. Case confirmation was done by RT-PCR. Passive surveillance data were monthly validated by the National System of Health Information (SNIS) following a defined process [25].

Environmental data was obtained from the National Meteorological Agency (ANAM) of Burkina Faso. Monthly aggregate environmental data consisted of: average relative humidity (in %), average minimum and maximum temperature (in °**C**), average rainfall (in mm), average wind speed (in m/s), average duration of insolation (in hour).

The population size at mid-year (demographic data) was extracted from the corresponding yearly Annuaire Statistique du Ministere de la Santé.

### Data analysis

Temporal patterns of dengue and environmental data between 2016 and 2021 were graphically represented monthly.

**Fig 1:**
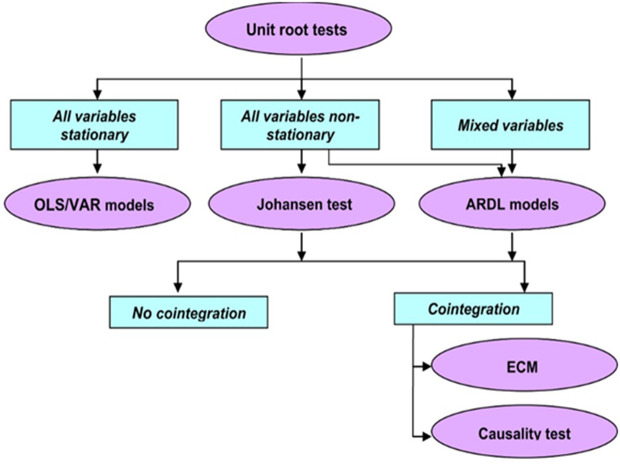
Steps taken in analyzing the data.

Dengue cases from 2022 to 2025 were forecasted and represented with a 95% confidence interval. The steps followed in analyzing the data are shown in Fig 1.

Then, the Augmented Ducker-Fuller test was used to test for a unit root in the times series. Some variables were stationary at a level, and others were not. The effect of environmental and demographic variables on dengue incidence was evaluated using the Autoregressive Distributed Lag (ARDL) model with the optimal lag, as we included stationary and non-stationary independent variables. The stability of the model was assessed through a diagnostic test and the recursive cumulative sum plot. No correlation was found in the model using the Lagrange-multiplier test. The normality of the different variables was confirmed with the Jarque-Bera normality test. The absence of multicollinearity in the model was tested by the variance inflation test.

## Results

### Temporal pattern of dengue between 2016 and 2021

**Fig 2:**
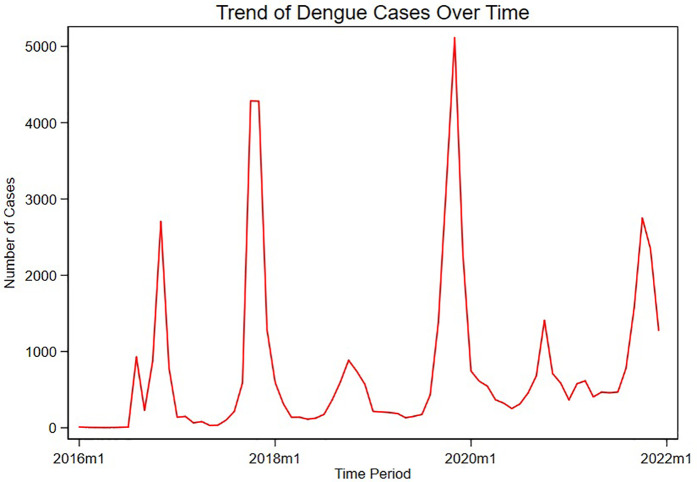
Time series pattern of dengue fever from 2016 to 2021.

Fig 2 shows the evolution of dengue from 2016 to 2021. There was a gradual increase in dengue cases over the study period. Furthermore, there are high spikes in specific periods based on seasonal variations. A critical look at the data also shows that dengue fever mostly spikes during the last quarter of every year. The highest spike in dengue cases occurred in late 2019, while the lowest cases of dengue were recorded in 2016.

### Temporal pattern of the climatic elements between 2016 and 2021

**Fig 3:**
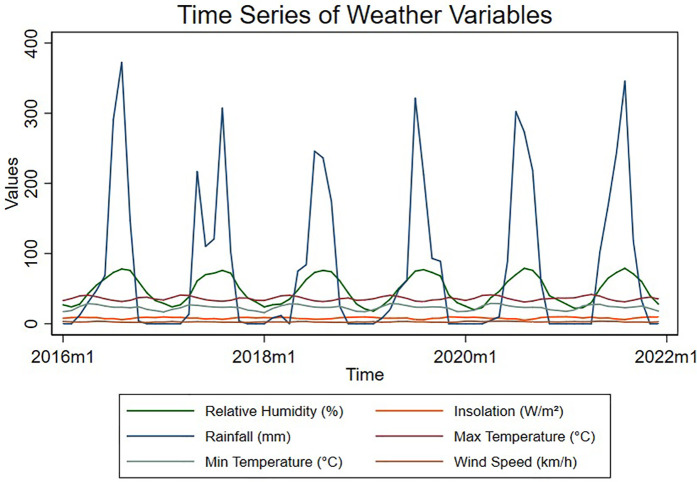
Time series graph of the environmental explanatory variables.

Fig 3 is a time-series graph that shows the nature of the environmental independent variables. A few independent variables are stationary, while others are nonstationary. However, an augmented Dickey-Fuller further examines the stationary properties of the study indicators.

### Assessing variables stationarity

Table 1 presents the results of the unit root test using an augmented Dickey-Fuller approach. Variables like dengue cases, population size, relative humidity, and rainfall are stationary at level. The rest of the variables are stationary at first difference.

**Table 1:** Augmented Dickey-Fuller test.

Variables	ADF at Level	ADF at First Difference	5% critical value
Dengue cases	−3.79*		−1.95
Population	2.34*		−1.95
Relative Humidity	−2.35*		−1.95
Insolation	−0.41	−5.59**	−1.95
Rainfall	−3.73*		−1.95
Maximum temperature	−0.60	−9.48**	−1.95
Minimum temperature	−0.86	−6.39**	−1.95
Wind speed	−0.59	−6.91**	−1.95

* Stationary at level.

** Stationary at first difference.

### Forecasting dengue incidence in the Central Region

**Fig 4:**
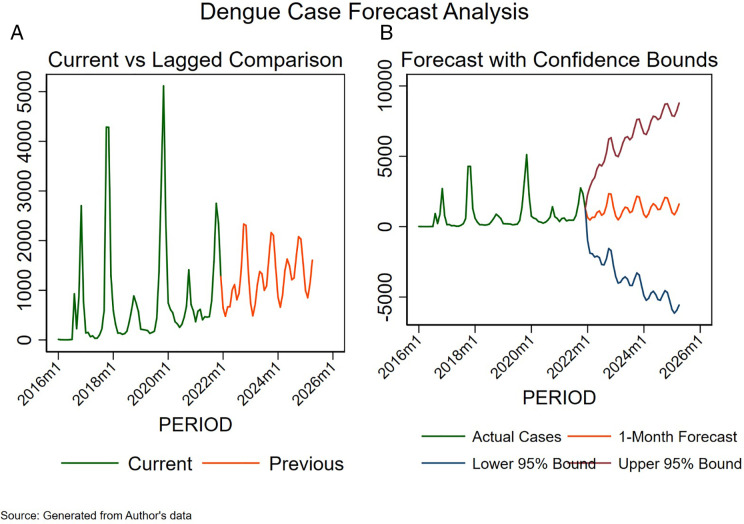
Three years point forecasts of dengue (A) and confidence interval (B) from 2022 to 2025.

Fig 4A and 4B forecast dengue cases from January 2022 to December 2025. The 95 per cent confidence interval widens and exceeds 5000 cases by 2023, reaching about 10,000 by 2025. However, the point estimation figures fall within the zero to five thousand range from 2022 to 2025. In Fig 4A, higher spikes of dengue cases are not seen from January 2022 to December 2025, as compared to the seasonal spikes observed in the past.

### Optimal lag selection

The lag selection criteria in Table 2 provide valuable insights into the optimal lag structure for the model. The Likelihood Ratio (LR) statistic is highest at lag 1 (723.47), indicating a strong fit, while the Final Prediction Error (FPE) is minimized at lag 4 (6.6e + 16), suggesting this lag may provide the best predictive performance. The Akaike Information Criterion (AIC) favors lag 4 with a value of 60.68, indicating that this model balances goodness of fit and complexity effectively. Similarly, both the Hannan-Quinn Information Criterion (HQIC) and Schwarz Bayesian Information Criterion (SBIC) also suggest lag 4 as optimal, with values of 64.12 and 69.37, respectively. Overall, the criteria collectively indicate that a lag of 4 is the most suitable choice for the model, ensuring both accuracy and efficiency in capturing the dynamics of the data.

**Table 2:** Lag selection criteria.

lag	LR	FPE	AIC	HQIC	SBIC
0		2.50E + 22	74.28	74.38	74.54
1	723.47	3.50E + 18	65.39	66.33	67.76*
2	268.21	4.70E + 17	63.30	65.07	67.77
3	190.83	2.30E + 17	62.36	64.96	68.94
4	240.43*	6.6e + 16*	60.68*	64.12*	69.37

# SBIC: Schwarz Bayesian information criterion.

### Effects of environmental factors on dengue incidence

In Table 3, the Autoregressive Lag Model (ARDL) results highlight several significant variables influencing dengue cases. The lagged variable Dengue cases (-1) shows a strong positive coefficient of 0.76 with a p-value of 0.00, indicating that previous dengue cases significantly affect current incidence. Additionally, the variable Dengue cases (-2) has a negative coefficient of -0.47 (p-value = 0.01), suggesting that cases from two lags ago negatively influence current cases. The variable Population of the region also statistically impacts dengue incidence with a coefficient of 0.00 and a p-value of 0.01, emphasizing the role of population size.

Among the environmental factors, Relative humidity (-1) positively affects dengue cases with a coefficient of 114.26 (p-value = 0.00), while Relative humidity (-4) also shows a significant positive effect (90.84, p-value = 0.00). Furthermore, Rainfall (-4) has a negative influence on dengue incidence with a coefficient of -6.91 and a p-value of 0.00. D.Minimum temperature (-3) has a positive coefficient of 223.20 with a p-value of 0.01, suggesting that minimum temperatures from three lags ago are associated with increased dengue incidence. Lastly, D.Wind speed shows a negative relationship with a coefficient of -925.31 (p-value = 0.02), indicating that higher wind speeds may reduce dengue cases, while D. Wind speed (-3) has a positive coefficient of 875.04 (p-value = 0.02).

**Table 3:** Autoregressive distributed lag model: ARDL (4,0,4,4,4,0,3,3).

Indicators	Coef.	Std. Err.	p-values	95% C. I
Dengue cases (-1)	0.76	0.13	0.00	0.5, 1.03
Dengue cases (-2)	-0.47	0.17	0.01	-0.81, -0.12
Dengue cases (-3)	0.43	0.19	0.04	0.03, 0.82
Dengue cases (-4)	-0.27	0.16	0.09	-0.59, 0.04
Population size_	0.00	0.00	0.01	<0.00, 01.0
Relative humidity	-38.91	27.11	0.16	-93.84, 16.01
Relative humidity (-1)	114.26	28.14	0.00	57.24, 171.28
Relative humidity (-2)	-31.96	32.56	0.33	-97.94, 34.02
Relative humidity (-3)	11.75	33.58	0.73	-56.28, 79.79
Relative humidity (-4)	90.84	27.10	0.00	35.93, 145.76
D.Insolation	-126.28	172.51	0.47	-475.82, 223.26
D.Insolation (-1)	-115.41	202.33	0.57	-525.37, 294.55
D.Insolation (-2)	-333.71	196.03	0.10	-730.91, 63.48
D.Insolation (-3)	-383.57	180.75	0.04	-749.81, -17.33
D.Insolation (-4)	-258.47	131.93	0.06	-525.79, 8.86
Rainfall	-3.66	2.55	0.16	-8.83, 1.51
Rainfall (-1)	-3.62	2.60	0.17	-8.88, 1.64
Rainfall (-2)	-3.62	2.40	0.14	-8.49, 1.24
Rainfall (-3)	-1.75	2.18	0.43	-6.17, 2.66
Rainfall (-4)	-6.91	2.10	0.00	-11.17, -2.66
D.Maximum temperature	-109.34	148.11	0.47	-409.45, 190.76
D.Minimum temperature	206.82	104.66	0.06	-5.23, 418.87
D.Minimum temperature (-1)	87.25	73.52	0.24	-61.72, 236.22
D.Minimum temperature (-2)	139.74	68.37	0.05	1.2, 278.27
D.Minimum temperature (-3)	223.20	74.35	0.01	72.54, 373.85
D.Wind speed	-925.31	361.75	0.02	-1658.29, -192.34
D.Wind speed (-1)	-292.78	418.28	0.49	-1140.3, 554.75
D.Wind speed (-2)	59.90	423.00	0.89	-797.18, 916.97
D.Wind speed (-3)	875.04	358.24	0.02	149.18, 1600.9
_cons	-8842.21	2351.64	0.00	-13607.07, -4077.34
***R-squared***	***0.85***			
***RMSE***	***537.4***			
***Adj R-squared***	***0.75***			

In Table 4, the ARDL long-run association results reveal significant relationships with dengue cases for several key indicators. Population size shows a positive association, with a coefficient of less than 0.001 and a p-value of 0.02, indicating that higher population size in the Central region correlates with increased dengue incidence. Relative humidity has a significant positive effect as well, with a coefficient of 264.99 and a p-value of 0.01. Additionally, Rainfall demonstrates a significant negative relationship, with a coefficient of -35.52 and a p-value of 0.04. Lastly, D.Minimum temperature shows a significant positive association with a coefficient of 1192.63 and a p-value of 0.02.

**Table 4:** ARDL long-run association.

Indicators	Coef.	Std. Err.	p-values	95% C. I
Population size	<0.001	<0.001	0.02*	<0001, < 0.001
Relative_humidity	264.99	96.93	0.01*	68.58, 461.4
D.Insolation	-2209.96	1320.72	0.10	-4885.99, 466.07
Rainfall	-35.52	16.56	0.04*	-69.07, -1.97
D.Maximum temperature_	-198.48	270.04	0.47	-745.64, 348.67
D.Minimum_temperature	1192.63	495.44	0.02*	188.76, 2196.5
D.Wind_speed	-513.99	2183.02	0.82	-4937.2, 3909.22

*Significant at 5%.

The bound test results in Table 5 indicate a significant long-run relationship among the variables. The F-statistic of 4.708 exceeds the upper critical bounds at all significance levels (1%: 4.26, 2.5%: 3.84, 5%: 3.50, 10%: 3.13), allowing us to reject the null hypothesis of no cointegration. Additionally, the t-statistic of -3.753 is more negative than the lower critical bounds at all levels (1%: -3.43, 2.5%: -3.13, 5%: -2.86, 10%: -2.57), further supporting the rejection of the null hypothesis and confirming cointegration. In summary, both statistics provide strong evidence of a significant long-run relationship among the variables, demonstrating their interconnectedness over time.

**Table 5:** Bound test.

Critical Values	F-statistics = 4.708		t-statistics = -3.753	
	Lower Bound	Upper Bound	Lower Bound	Upper Bound
1%	2.96	4.26	-3.43	-5.19
2.5%	2.60	3.84	-3.13	-4.85
5%	2.32	3.50	-2.86	-4.57
10%	2.03	3.13	-2.57	-4.23

Statistical significance = 0.01 - 0.1, k = 8 regressors.

## Discussion

This study aimed to forecast dengue incidence between 2022 and 2025 in the Central Region, Burkina Faso. There was a gradual increase in dengue cases over the study period (2016–2021), with the lowest number of cases in 2016 and the highest in 2019, as already reported in the same Region [25]. Despite warnings of the disease in the 2000s, it took the 2013 epidemic for dengue surveillance to be set up in the Central Region [26,27]. Passive surveillance of dengue began in the Central Region in 2014. Under this scheme, cases report has been improved since 2016. Dengue cases have been consistently underreported in Africa and misdiagnosed as malaria, due to poor surveillance and the unavailability of diagnostic tools [7].

Using only the historical data, a gradual increase in dengue cases was forecasted between 2022 and 2025. Dengue cases were then expected to rise between 2022 and 2025, with point estimates of about 5000 cases. The 95% confidence interval (CI) exceeded already 5000 in 2023, and would almost reach 10,000 by 2025. The projected increase of cases was confirmed in 2023, as Burkina Faso faced the largest epidemic of dengue ever recorded in Africa. In 2023, the country reported 154,867 suspected cases, 70,433 probable cases and 709 deaths [28]. From epidemic week 1 in 2023 to epidemic week 17 in 2024, the WHO Afro region reported 241, 226 cumulative cases of dengue and 874 cumulative deaths due to DENV-1, DENV-2 and DENV-3 in 15 WHO African countries out of 47 [5]. The year 2023 was epidemiologically exceptional for countries like Bangladesh, Pakistan, Brazil, Fiji, the Philippines, Vietnam and Burkina Faso [29]. Changes in weather patterns due to climate change, changes in the geographical distribution of *Aedes aegypti* in previously non endemic settings, unplanned urbanization in the different cities [29], co-circulation of dengue serotypes, and shift in the dominant serotype over time [30] could explain the global alarming situation of dengue. In particular, El Niño-Southern Oscillation (ENSO), which constitutes a significant risk for dengue, has also worsened the global dengue situation in 2023, intervening in subsequent years [29,31].

Climatic factors like rainfall, temperature and relative humidity are the most significant predictors of dengue cases, dengue incidence rate and dengue outbreaks [15,19,20]. In effect, climatic elements impact indirectly dengue transmission by interfering the biological mechanisms of the *Aedes* mosquitoes, with potential lags [32]. Climatic predictors have been in all predicting models [19,20]. With time series, autoregressive models are the most widely used models (26.7%) to predict dengue incidence [19,20]. Autoregressive Distributed Lag (ARDL) model offers the dual advantage of capturing short-term dynamics, while estimating long-term equilibria between variables. In fact, the ARDL analyses pointed the multifaced influences of environmental effects in this study. While previous dengue cases 1 lag ago significantly affect current incidence, dengue cases 2 lags ago negatively affect current cases. The role of population size is also emphasized in this study as it statistically impacts dengue incidence. In the long run perspective, the Central Region population correlates with increased dengue incidence long run relationship. With a growth rate of 4.4%, the population of the Central Region has passed from 1,727,290 inhabitants in 2006–3,030,384 inhabitants in 2019 [23]. Given this growth rate, dengue cases are expected to increase in the coming years. Although not frequently used in the prediction models of dengue, demographic factors are sometimes associated with climate and climate change factors (3.1%) or with only climate elements (5.2%) [19].

Among the environmental factors, Minimum temperature (-3) three lags ago is associated with increased dengue incidence. In the long run relationship, minimum temperature shows a significant positive association, suggesting that higher minimum temperatures contribute to increased dengue cases. Temperature is crucial in dengue transmission as it influences the survival and metabolic processes of mosquitoes, subsequently their competence, and dengue virus proliferation within adult *Aedes* known as the extrinsic incubation [33]. To be effective, the effect of temperature must remain within specific limits. Thus, the optimal minimum and maximum temperatures were 14.88°C (95% CI 12.4-16.68°C) and 32°C -33°C, respectively [34,35]. For the development of adult *Aedes* mosquitoes, the optimal temperature lay between 25°C and 30°C [33]. Outside these limits, specifically beyond the higher limits, adult *Aedes* would die, and the larvae and egg would fail to evolve. In the Central Region of Burkina Faso, the optimal minimum temperature for dengue was 18°C to 20°C, and the maximum temperature was 27°C to 32°C established from data covering the period 2017–2019 [21].

Aside from minimum temperature, maximum temperature, relative humidity, and wind speed were significantly associated with dengue in Burkina Faso [21]. The ARDL model found a negative relationship between Rainfall and dengue incidence, while it was expected to favor dengue transmission by providing more breeding sites for mosquitoes. Similarly, the long run association triggered a significant negative relationship of dengue cases with rainfall, indicating that certain rainfall conditions may reduce mosquitoes habitats, essential for the survival of immature mosquitoes and the reproduction of adults [36]. In fact, heavy rain or floods could destroy *Aedes* breeding sites and eggs and endanger dengue virus transmission.

Relative humidity, 1 and 4 lags ago positively affect dengue cases, as found in the long run relationship. Indeed, relative humidity is a key factor in the extrinsic incubation of dengue virus into the vector, but also in the activities of adult mosquitoes, suggesting that increased humidity levels enhance mosquito proliferation, thereby raising dengue transmission rates. The aforementioned effect of ambient temperature on the survival and reproductive activity of female Aedes is modulated by relative humidity [37]. Rarely evaluated, higher Wind speed without any lag may reduce dengue cases, while Wind speed (-3) has a positive influence on dengue incidence, suggesting a complex relationship over time. These findings underscore the multifaceted influences of climatic factors on dengue transmission dynamics.

### Limitation of the model

The use of the Autoregressive Distributed Lag (ARDL) model to analyze the effects of environmental factors on dengue fever incidence has some limitations, primarily due to the inclusion of only a few predictors such as population size and weather variables. This limited parameterization may overlook critical factors like socio-economic conditions and healthcare access. Additionally, the model assumes linear relationships and constant dynamics over time, which may not reflect the complexities of epidemiological data. In future studies, adopting a multiparametric approach that includes additional predictors, employing nonlinear modeling techniques, ensuring robust data collection, and accounting for confounding variables will enhance the analysis. These improvements will provide a more comprehensive understanding of the factors influencing dengue incidence and inform more effective public health strategies.

## Conclusion

Dengue is endemic in the Central Region of Burkina Faso, with cases reported every month from 2016 to 2021. Using epidemiological and environmental data, we forecasted dengue incidence between 2022 and 2025. The disease was predicted to remain a serious menace for the country, as cases are expected to rise between 2022 and 2025. Burkina Faso should tailor a long-term response to the dengue to be integrated with the interventions for malaria control. These interventions should also consider the potential interactions of dengue with other neglected diseases like chikungunya and Zika disease already found by seroepidemiological studies.

## Supporting information

S1 Fig Stability test of the model. (TIF)

S2 Fig Results of the cumulative sum test. (TIF)

S3 Fig Three years point forecasts of dengue and confidence interval from 2022 to 2025. (TIF)

S1 Table Autocorrelation test. (DOCX)

S2 Table Jarque-Bera normality test. (DOCX)

S3 Table Multicollinearity test. (DOCX)

S4 Table Heteroskedasticity test. (DOCX)

S1 File Revised database. (XLS)

S2 File Supplementary information. (DOCX)
