# Supplementary figures and images for: Effects of environmental factors on dengue incidence in the Central Region, Burkina Faso: A time series analyses
Source: PLoS Negl Trop Dis. 2025 Jul 28;19(7):e0013356. doi: 10.1371/journal.pntd.0013356 (PMC12313059; doi:10.1371/journal.pntd.0013356)

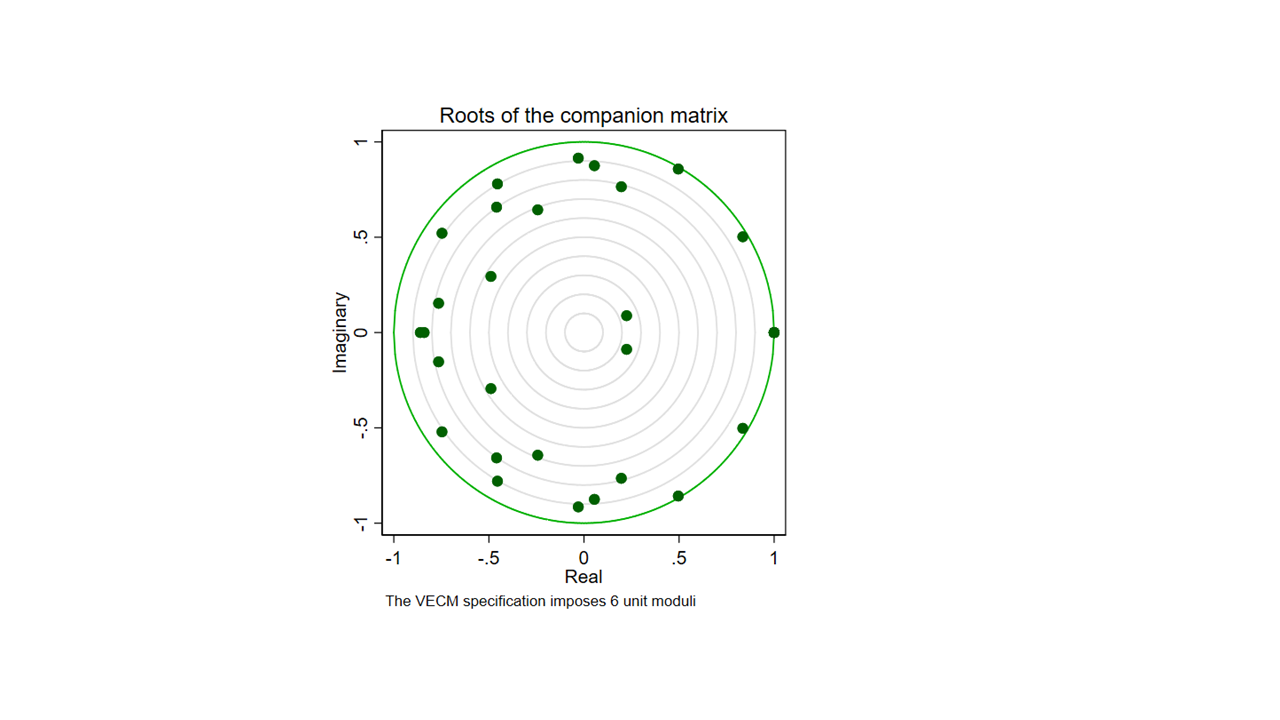

Supplement: S1 Fig — (TIF) [file pntd.0013356.s001.tif]

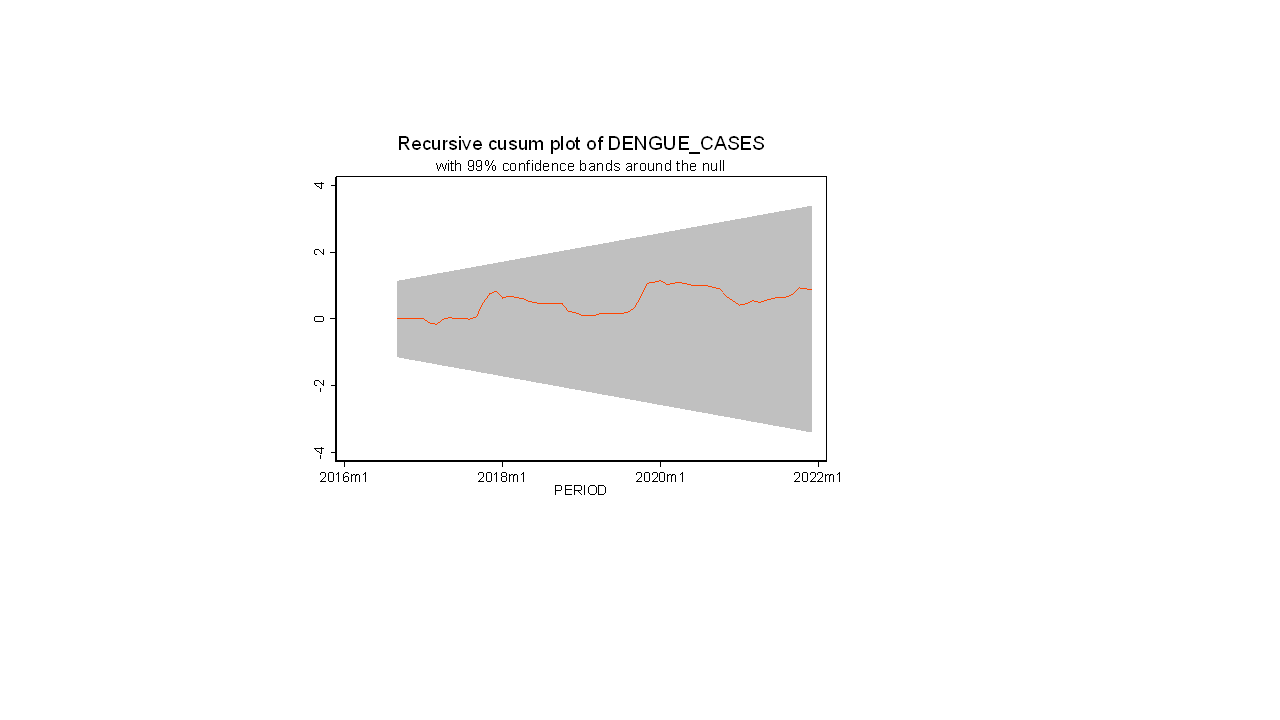

Supplement: S2 Fig — (TIF) [file pntd.0013356.s002.tif]

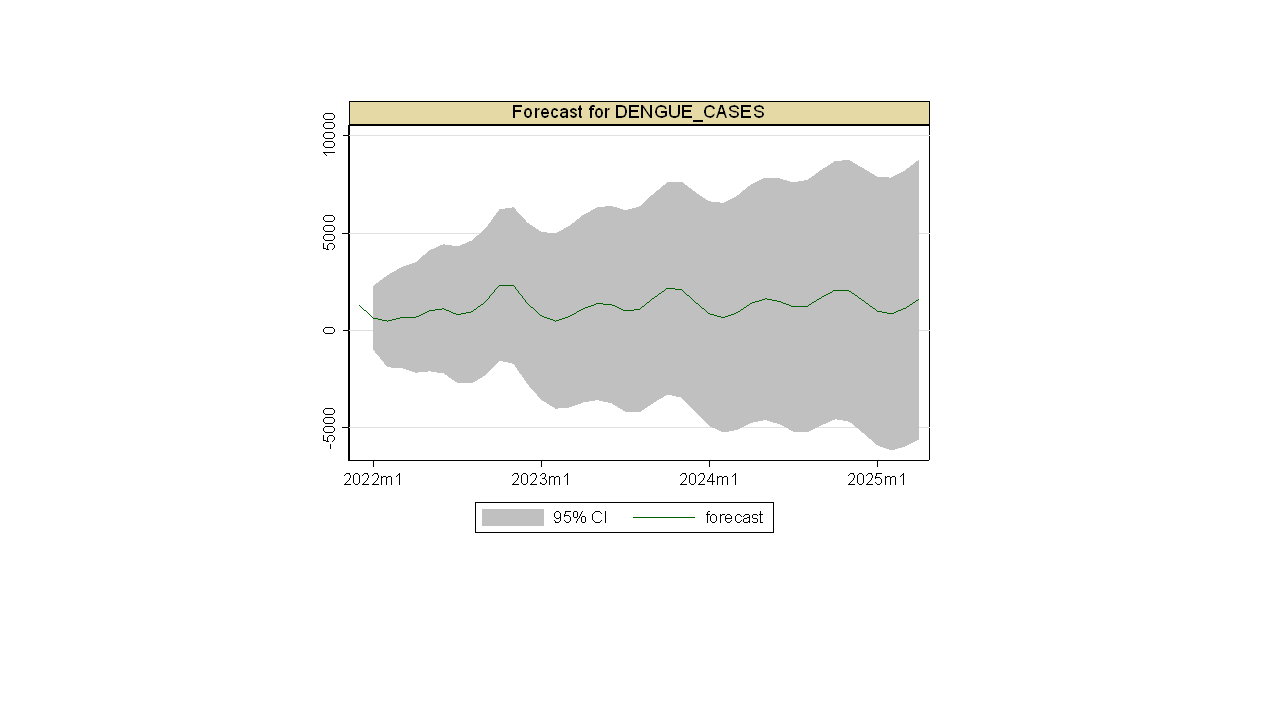

Supplement: S3 Fig — (TIF) [file pntd.0013356.s003.tif]
