# Supplementary material for: Effects of environmental factors on dengue incidence in the Central Region, Burkina Faso: A time series analyses
Source: PLoS Negl Trop Dis. 2025 Jul 28;19(7):e0013356. doi: 10.1371/journal.pntd.0013356 (PMC12313059; doi:10.1371/journal.pntd.0013356)
Supplement: S1 Table — (DOCX) [file pntd.0013356.s004.docx]

| lag | chi2 | df | p-values |
| --- | --- | --- | --- |
| 1 | 90.32 | 64.00 | 0.02 |
| 2 | 81.57 | 64.00 | 0.07 |
| 3 | 58.47 | 64.00 | 0.67 |
| 4 | 74.10 | 64.00 | 0.18 |

**S1 Table: Autocorrelation test**
