# Supplementary material for: Effects of environmental factors on dengue incidence in the Central Region, Burkina Faso: A time series analyses
Source: PLoS Negl Trop Dis. 2025 Jul 28;19(7):e0013356. doi: 10.1371/journal.pntd.0013356 (PMC12313059; doi:10.1371/journal.pntd.0013356)
Supplement: S2 Table — (DOCX) [file pntd.0013356.s005.docx]

**S2 Table: Jarque-Bera Normality Test**

| **Equation** | **Chi2** | **df** | **p-values** |
| --- | --- | --- | --- |
| D.Dengue cases | 51.81 | 2.00 | 0.00 |
| D.Population size | 0.75 | 2.00 | 0.69 |
| D.Relative humidity | 0.25 | 2.00 | 0.88 |
| D2.Insolation | 1.70 | 2.00 | 0.43 |
| D.Rainfall | 1.37 | 2.00 | 0.50 |
| D2.Maximum temperature | 1.57 | 2.00 | 0.46 |
| D2.Minimum temperature | 12.82 | 2.00 | 0.00 |
| D2.Wind speed | 1.26 | 2.00 | 0.53 |
| ALL | 71.52 | 16.00 | 0.00 |
