# Supplementary material for: Effects of environmental factors on dengue incidence in the Central Region, Burkina Faso: A time series analyses
Source: PLoS Negl Trop Dis. 2025 Jul 28;19(7):e0013356. doi: 10.1371/journal.pntd.0013356 (PMC12313059; doi:10.1371/journal.pntd.0013356)
Supplement: S3 Table — (DOCX) [file pntd.0013356.s006.docx]

**S3 Table Multicollinearity Test**

| Variable | VIF |
| --- | --- |
| D.Maximum temperature | 5.53 |
| D.Minimum temperature | 3.07 |
| D.Insolation | 2.22 |
| Rainfall | 2.15 |
| D.Wind speed | 1.94 |
| Population size | 1.00 |
| Mean VIF | 2.65 |
