# Supplementary material for: Effects of environmental factors on dengue incidence in the Central Region, Burkina Faso: A time series analyses
Source: PLoS Negl Trop Dis. 2025 Jul 28;19(7):e0013356. doi: 10.1371/journal.pntd.0013356 (PMC12313059; doi:10.1371/journal.pntd.0013356)
Supplement: S4 Table — (DOCX) [file pntd.0013356.s007.docx]

**S4 Table Heteroskedasticity test**

| Source | chi2 | df | p-values |
| --- | --- | --- | --- |
| Heteroskedasticity | 36.44 | 27.00 | 0.11 |
