# Supplementary material for: Effects of environmental factors on dengue incidence in the Central Region, Burkina Faso: A time series analyses
Source: PLoS Negl Trop Dis. 2025 Jul 28;19(7):e0013356. doi: 10.1371/journal.pntd.0013356 (PMC12313059; doi:10.1371/journal.pntd.0013356)
Supplement: S2 File — (DOCX) [file pntd.0013356.s009.docx]

**SUPPLEMENTARY FILES**

**Diagnostic test**

Fig 1 illustrates that all of the roots fall inside the circle, without spreading beyond its limits. The results suggest that the estimated ARDL model is stable and will consistently and accurately anticipate the growth rate over time. The findings are considered valid and reliable since all data points are contained inside the boundaries of the circle.

**S1 Fig: Stability test of the model**

**Autocorrelation test**

The autocorrelation test results reveal that there is significant autocorrelation in the residuals at lag 1, as indicated by a chi-squared value of 90.32 and a p-value of 0.02. This suggests that residuals are correlated over this time period, which could impact the validity of the regression model. At lag 2, the p-value of 0.07 is close to the conventional threshold, indicating a potential autocorrelation issue that may warrant further investigation, although it is not statistically significant. Conversely, lags 3 and 4 show high p-values (0.67 and 0.18, respectively), indicating no significant autocorrelation.

**S1 Table: Autocorrelation test**

**Test of a parameter stability**

Fig 2 represents a cumulative sum test, used to assess the stability of a parameter. The red line represents the total amount of values. If the red line falls inside the shaded region, the test will fail to reject the null hypothesis at the 5 per cent significance level. The graph representing the research is located inside the area that is shaded. This implies that there is no structure break in the model. Therefore, the model accurately monitors the rate of occurrence in dengue cases. The coefficients of the ARDL model exhibit statistical stability over time.

**S2 Fig: Results of the cumulative sum test**

**Normality test of the distribution**

The Ordinary Least Squares (OLS) approach relies on the fundamental assumption that the data follows a normal distribution. The variables included in the regression model must adhere to a normal distribution. The study used the Jarque-Bera test for analysis. S2 Table reveals that while some variables (population, relative humidity, insolation, rainfall, and wind speed) approximate a normal distribution, others, particularly dengue cases and minimum temperature, exhibit significant skewness.

**S2 Table: Jarque-Bera Normality Test**

**Multicolinearity test**

The Variance Inflation Factor (VIF) results indicate varying levels of multicollinearity among the independent variables in the regression model. D.Maximum temperature has a VIF of 5.53, suggesting moderate multicollinearity. In contrast, D.Minimum temperature (3.07), while also showing some multicollinearity, remains below the concern threshold of 5. Other variables, such as D.Insolation (2.22), RAINFALL (2.15), and D.Wind speed (1.94), exhibit low multicollinearity, indicating their stability as predictors. With Population size showing a VIF of 1.00, it is independent of the other variables. The mean VIF of 2.65 suggests that, overall, multicollinearity is not a significant issue.

**S3 Table Multicollinearity Test**

**Heteroskedasticity test**

The p-value is greater than 0.05, indicating that there is no significant evidence of heteroskedasticity in the model. This suggests that the variance of the residuals is constant across observations, which is a desirable property for regression analysis.

**S4 Table Heteroskedasticity test**

**Dengue forecasts and 95% confidence interval**

**S3 Fig: Three years point forecasts of dengue and confidence interval from 2022 to 2025**
